# A Compact and Robust RFID Tag Based on an AMC Structure

**DOI:** 10.3390/s24051468

**Published:** 2024-02-24

**Authors:** Giovanni Andrea Casula, Giacomo Muntoni, Paolo Maxia, Giorgio Montisci

**Affiliations:** 1 Department of Electrical and Electronic Engineering, University of Cagliari, 09123 Cagliari, Italy; giacomo.muntoni@unica.it (G.M.); giorgio.montisci@unica.it (G.M.); 2 INAF—Osservatorio Astronomico di Cagliari, 09047 Selargius, Italy; paolo.maxia@inaf.it

**Keywords:** wearable antennas, RFID tags, human body coupling, AMC structures

## Abstract

A platform-tolerant RFID (Radio-Frequency Identification) tag is presented, designed to operate across the entire RFID band. This tag utilizes a small Artificial Magnetic Conductor (AMC) structure as a shielding element for an ungrounded RFID tag antenna. It can be easily mounted on various surfaces, including low permittivity dielectric materials, metal objects, or even attached to the human body for wearable applications. The key features of this RFID tag include its ability to be tuned within the worldwide RFID band, achieving a maximum theoretical read range of over 11 m. Despite its advanced capabilities, the design emphasizes simplicity and cost-effective manufacturing. The design and simulations were conducted using CST Studio Suite.

## 1. Introduction

The primary challenge in designing wearable antennas arises from the strong coupling between the antenna and the human body. The human body, being a lossy and non-homogeneous material, can significantly degrade antenna performance when compared to free-space applications [1].

Considering as a reference the stand-alone antenna, the coupling between the antenna and the human body modifies the input impedance, causes a resonant frequency detuning, and is responsible for the radiation efficiency degradation and for the radiation pattern fragmentation. All these effects are strictly related to the antenna size, layout, and operating frequency [1,2]. Moreover, in real-world applications, the antenna–body distance randomly changes due to natural movements of the wearer [2], and the geometrical and electromagnetic parameters of the antenna platform (the human body) typically change from person to person and vary also considering different locations of the antenna on the same person (i.e., head, chest, leg, arm) [3]. Furthermore, the antenna behavior is strongly dependent on its distance from the human body surface. Therefore, in wearable applications, the effect of the human body on system performance must be adequately limited and shielded, resorting to appropriate design choices which aim to improve the antenna’s robustness.

At the UHF band, antennas with a size between λ/5 and λ/2 (λ being the free-space wavelength) are quite common, since they allow for the realization of a relatively efficient wearable antenna that can still be made unobtrusive and comfortable to the user. 

Wearable antennas without the ground plane [4] (denoted in the following as “ungrounded” antennas) can be directly integrated in existing clothes, also thanks to the recent advances in textile antennas design and in fabrication of circuits with intricate details [5,6]. However, the absence of a metallic ground plane makes these antennas extremely sensitive to the human body proximity effect. Thus, increasing their robustness with respect to the antenna–body coupling effects is an important challenge for the antenna designer. In [7], the concepts described in [8,9,10,11,12] have been extended to improve the robustness of “ungrounded” printed wearable antennas. Despite this improvement, the efficiency of these antennas is lower than 10% when they operate attached to the body, or within a couple of millimeters from the wearer, which is the most common situation.

Considering the low efficiency of ungrounded wearable antennas, the most used configuration for a wearable antenna consists of a grounded structure, with the ground plane (which should be as small as possible) printed on the back side of the dielectric layer. In fact, the use of such a grounded structure is an easy and effective solution which minimizes the coupling effects between the antenna and the human body. On the other hand, a large ground plane is in strong contrast with the most important features requested in wearable antennas, since the physical structure of grounded antennas limits mechanical flexibility, comfort, and wearability. 

A criterion to guide the designer in the choice of the optimal shape and size of the antenna ground plane to obtain an improvement in the robustness of UHF grounded wearable printed antennas has been proposed in [8,9,10,11,12]. This criterion helps to design a ground plane as small as possible, limiting the antenna size, and relates the optimal ground plane shape to the position of the maxima of the electric and magnetic energy density distributions in the near-field region around the antenna. The degradation of antenna performance caused by human body coupling can be reduced if the ground plane is modified, aiming to confine the electric energy density in the region far from the antenna border. Therefore, a grounded wearable antenna with a minimal impact on the comfort of the wearer can be effectively designed.

An alternative and most effective configuration with respect to grounded structures can be used to obtain a higher performing wearable antenna. This solution, described in this work, consists of using Artificial Magnetic Conductors (AMCs), which can be exploited by the designer to preserve the antenna radiation characteristics and performance, thanks to their capability of isolating an antenna from the surrounding environment [13,14,15].

This paper describes the design of a relatively small AMC structure used as a shielding element for an ungrounded RFID tag antenna at UHF frequencies selected from the open literature, namely the nested-slot suspended patch (NSSP) antenna, a slot aperture antenna operating at 868 MHz, whose performance has been assessed and experimentally tested in [4]. The performance of this antenna is compared when it is attached to the human body or placed upon an “ad-hoc” designed AMC structure, which shields it from the wearer. As a result, the proposed structure presents a very good robustness and isolation with respect to the same antenna without the integration of AMCs. Moreover, the NSSP antenna gain increases about 13 dB, due to the isolation provided by the AMC planar structure, showing a significant improvement in the wearable antenna’s performance. Moreover, the matching between the NSSP and its microchip is easy to perform if compared with the case of NSSP directly attached to the human body.

The designed wearable UHF RFID tag can be effectively used also as an on-metal tag and as a platform-tolerant tag, due to the presence of the AMC structure, which allows the tag to have high isolation from the working environment, with a reading range beyond 5.5 m on low permittivity dielectric materials, 8 m when attached to the human body, and 11 m on a 200 × 200 mm^2^ metal plate. 

In Table 1, the proposed tag antenna has been compared with state-of-the-art works dealing with tags on AMCs [16,17,18,19,20], metal tags ([16,19,20,21,22,23,24,25,26]), and platform-tolerant tags ([22,23,25]).

The tags on AMCs [16,17,18,20] are significantly larger than the proposed structure, while [19] is almost 40% smaller, but has definitely the worst performance. Moreover, these tags work on metal ([16,19,20]) or on the human body ([17,18]), whereas our proposed structure exhibits a similar behavior both when mounted on metal objects and when worn by a user.

The dual-polarized dual-planar inverted-F tag antenna with polarization diversity proposed in [22] can achieve a good read range, up to 10.2 m, but it involves both lumped elements and 12 vias, resulting complex and expensive structures to realize. Moreover, the structure is very sensitive to these vias, since its resonant frequency highly depends on their positioning. 

The dipolar patch tag presented in [23] has a small footprint and an orientation insensitive capability, and it is particularly suitable for platform-tolerant or metal-mountable applications. On the other hand, the relatively small value of the real part of its input impedance (around 4 Ω) severely limits its working frequency bandwidth, causing a poor matching with the RFID chip. In addition, the reading range of this tag is limited to only 3.5 m on metal and less than 2 m on dielectric materials. 

The metal-mountable folded cross-dipole-based tag described in [25] has a relatively small size and polarization diversity features, but the parasitic metal ring placed beneath the radiator, used to tune down the resonant frequency, makes this design a bit complex, and its read range varies from 5.6 m to 7.7 m depending on the structure of the metal objects on which it is mounted.

Similarly to [23], the cross-dipole based antennas presented in [22,25] have a small 3 dB bandwidth caused by the very low real part of their input impedance, and this can limit their applications as platform-tolerant and on-metal antennas.

The tag antenna described in this paper has also been compared with circularly polarized cross-dipole-based tag antennas [27,28,29,30], having small size and good read range performances. Such antennas have typically a single-layer substrate without a ground plane; therefore, they cannot be used in wearable, metal-mountable, and platform-tolerant applications. 

The proposed RFID structure has been designed using CST Microwave Studio 2023, a general-purpose software for the 3D electromagnetic simulation of microwave components. CST is a well-assessed and established electromagnetic software for more than 20 years, and its results can be considered equivalent to experimental data, as reported in the open literature for a wide range of applications (see, for example, [7,8,9,10,11,12,31,32,33,34,35,36,37]). Hence, we rely on CST results for assessing the performance of the proposed structure.

## 2. AMC Design

An AMC is a frequency-selective surface, a periodic structure showing a band-pass or band-stop characteristic at a fixed frequency, and is typically made of conductive elements printed on a grounded dielectric substrate. The AMC structures are artificially constructed surfaces having electromagnetic properties that do not exist in nature. 

There are various types of metamaterials used to implement an AMC structure, such as Electromagnetic Bandgap (EBG) [38,39], Electric-LC (ELC) [40], Double-Negative material (DNG)/Double-Positive material (DPS) [41], Split Ring Resonators (SRR) [42], Photonic Band Gap (PBG) materials [43], metamaterial absorbers [44], and metamaterial beams [45]. Metamaterial incorporation into microwave structures provides flexibility in manipulating the electromagnetic behavior of the antenna and of microwave devices in general [46]. In contrast to Perfect Electric Conductors (PECs), an AMC generates reflected waves that are similar in the direction of the original current, with the reflection coefficient Γ equal to +1 (instead of −1, as in the case of PEC). As a result, the wave reflected by an AMC is in-phase with the source wave, and constructively interferes with it. The combining effect from both reflected waves and source waves improves the antenna radiation efficiency and gain, allowing one to design a low-profile antenna without adding an unnecessary distance of λ/4 between the AMC ground plane and the antenna. This spacing, which is required for structures with a PEC ground layer to improve their performance, would represent an unacceptable thickness for UHF wearable antennas. 

Since the principle of AMC operation is based on the resonance of the cavity between the periodic elements and the ground plane, AMC structures act like a Perfect Magnetic Conductor (PMC) only within a limited frequency band and are therefore narrowband structures (as can be deduced by the steeper gradient of their frequency response in terms of the phase of the reflection coefficient Γ). The bandwidth of an AMC structure can be defined as the frequency interval where the phase of the reflection coefficient Γ is comprised between +90° (lower frequency, f_L_) and −90° (higher frequency, f_H_), with 0° as the designated resonant frequency, f_R_. In the remaining band (below f_L_ and beyond f_H_), the AMC acts like a PEC [47]. Since the AMC reflects the reflected wave in-phase with the source wave, it significantly enhances the front-to-back ratio and consequently reduces the SAR, while maintaining a relatively large impedance bandwidth.

The shape of the periodic elements constituting the unit cells of an AMC structure can be very different, and ranges from simple (dipoles, patches) to complex geometries [48], but, in any case, the physical size of the unit cell must be close to half a wavelength, due to the AMC’s resonant behavior. 

By employing an AMC, it is possible to place a radiating element close (or attached) to the meta-surface without degrading its performance, since the AMC restricts the propagation of surface waves within a specific frequency band (known as band gap) and therefore reduces the level of unwanted back-lobe radiations toward the human body, having only a negligible effect on the radiating properties of the whole structure. This could be very important in wearable applications, where the smart use of AMC structures can be exploited to obtain high robustness and isolation between the antenna and its platform (the human body). 

Unfortunately, AMC structures can be cumbersome, especially in the lower part of the UHF frequency band (where even half a wavelength can be an unpractical size in several applications); therefore, an extremely challenging aspect in AMC design is to minimize the size of the periodic unit cell constituting the AMC to better approximate a homogenous medium. 

The simplest way to obtain a significant space reduction (which approximately is inversely proportional to ε_r_, with ε_r_ being the dielectric permittivity of the substrate) is the use of high permittivity dielectric materials as substrates for the AMC structure, although they have relatively high losses and can be expensive. In this work, we choose ARLON (ε_r_ = 6, tanδ = 0.0004) to implement both the AMC and tag. This substrate can allow an adequate size reduction of about 60% if compared to the case of a substrate with ε_r_ = 1, obtaining a compact and comfortable structure. The substrate thickness is equal to 1.57 mm in both cases, so as to limit the vertical size of the complete structure (Tag + AMC thickness is 3.245 mm, corresponding to 0.009 λ_0_ at the design frequency).

**Figure 1:**
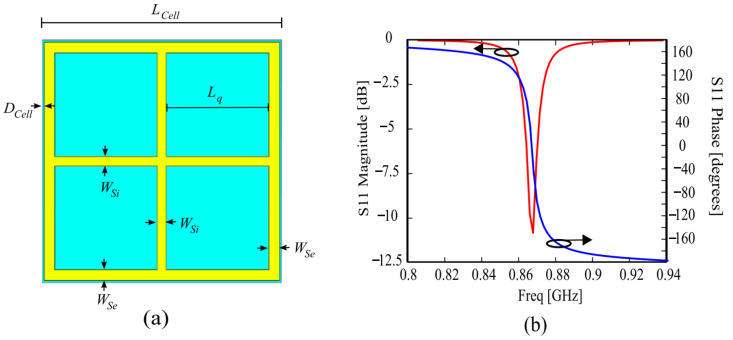
(**a**) Geometry of the proposed AMC unit cell; (**b**) Magnitude and phase of the reflection coefficient S_11_ of the unit cell. L_Cell_ = 47.35 mm, L_q_ = 20.25 mm, D_Cell_ = 0.4 mm, W_Si_ = 1.85 mm, W_Se_ = 2.1 mm.

The AMC unit cell has been designed to resonate at the RFID UHF European frequency of 868 MHz, and its geometry is shown in Figure 1a. The main parameter of the cell is the intercell distance D_Cell_, which can be chosen to tune the resonant frequency of the periodic structure. In particular, the resonant frequency can be lowered by decreasing D_Cell_. On the other hand, D_Cell_ should not be too small, in order to avoid a strong coupling between the AMC adjacent cells. The design value of D_Cell_ is 0.4 mm, and the AMC unit cell has a periodicity of D = 47.35 mm (0.136 λ_0_ at 868 MHz). 

The reflection coefficient S_11_ of the AMC structure for an incident plane wave is reported in Figure 1b in both magnitude and phase.

**Figure 2:**
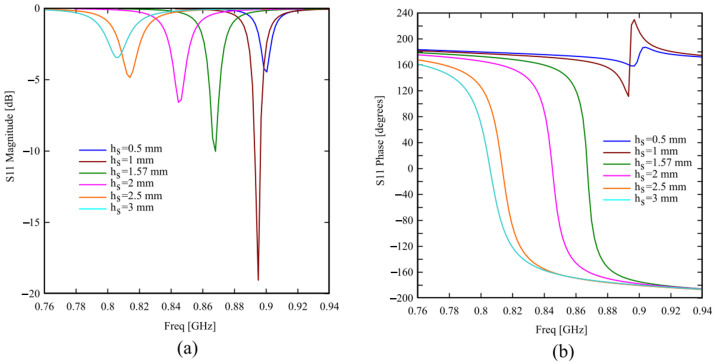
(**a**) Magnitude and (**b**) phase of the reflection coefficient S_11_ of the unit cell for different substrate thicknesses h_s_.

In Figure 2, the magnitude and phase of the reflection coefficient S_11_ is reported for different substrate thicknesses, showing that the AMC behavior and bandwidth are better for thick substrates; therefore, the designer must choose an adequate compromise between the AMC structure profile and its performance. 

**Figure 3:**
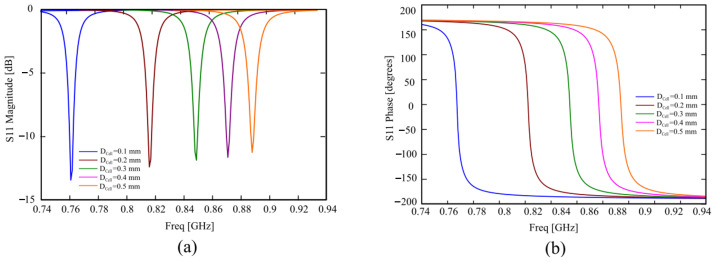
(**a**) Magnitude and (**b**) phase of the reflection coefficient S_11_ of the unit cell for different substrate thicknesses D_cell_.

Figure 3 shows the magnitude and phase of the reflection coefficient S_11_ for different intercell distances. In this case, the behavior is substantially the same, but smaller values of the intercell distance can shift the resonance toward lower frequencies, thus reducing the cell size.

## 3. Results and Comparison

**Figure 4:**
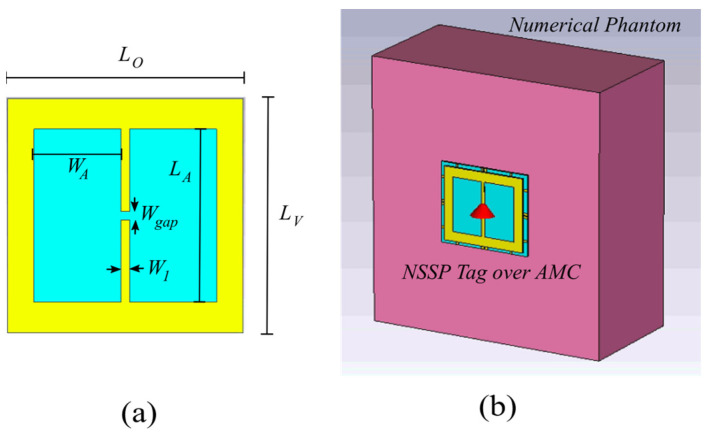
(**a**) Layout of the NSSP ungrounded tag antenna. W_A_ = 25.65 mm, L_A_ = 51.3 mm, W_gap_ = 2.56 mm, W_1_ = 2.56 mm, L_O_ = L_V_ = 69.44 mm; (**b**) Designed tag antenna over the AMC structure attached on the single-layer muscle-like phantom model used to perform the numerical investigation of the antenna’s robustness to the body proximity.

To demonstrate the advantages of using the AMC structure to isolate the tag from the human body, we consider the single-layer slot antenna for UHF RFID tags proposed in [4], operating at 868 MHz. This antenna is called nested-slot suspended patch (NSSP) and consists of an H-shaped slot placed onto a suspended patch. The antenna layout is shown in Figure 4a. This is a versatile layout, because it is capable of matching a large class of microchips by a suitable choice of its geometrical parameters [4]. The tag antenna is etched on a rectangular metallic plate printed on a 1.57 mm thick Arlon dielectric slab (ε_r_ = 6, tanδ = 0.0004), which electrically insulates the antenna from the body. We use an Impinj Monza 4 microchip for the NSSP tag, with an input impedance Z_chip_ equal to 13-j151 Ω at 868 MHz.

To account for the presence of the human body, a numerical phantom has been added to the simulation scenario (see Figure 4b). It is a single-layer muscle-like equivalent model consisting of a material with size 20 × 20 × 10 cm^3^ and with ε_r_ =2/3, ε_r_muscle_ = 36, σ = 2/3, and σ_muscle_ = 0.62 S/m [7]. The reported simulations have been performed with CST Microwave Studio.

**Figure 5:**
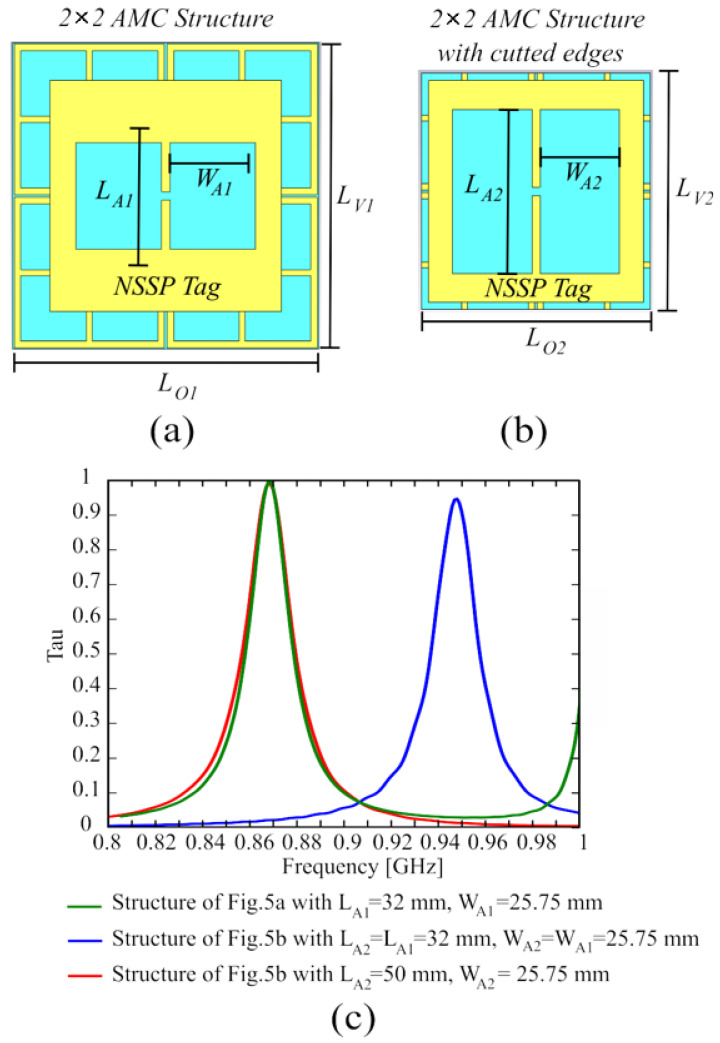
(**a**) Layout of the NSSP ungrounded tag antenna on the 2 × 2 AMC structure: L_O1_ = 94.70 mm, L_V1_ = 94.70 mm, L_A1_ = 32 mm, W_A1_ = 25.65 mm; (**b**) Reduced 2 × 2 AMC structure after cutting the outer edges: L_O2_ = 76 mm, L_V2_ = 76 mm, L_A2_ = 50 mm, W_A2_ = 25.65 mm. (**c**) Transmission coefficient for the configurations shown in (**a**,**b**).

The finite AMC screen placed underneath the tag antenna is composed of an array of 2 × 2 unit cells (Figure 5a), which represents the smallest size for a finite AMC structure; hence, the composed radiator has a very compact size of 94.7 × 94.7 mm^2^ (0.272∙λ_0_ × 0.272∙λ_0_, where λ_0_ =345.62 mm is the free-space wavelength at 868 MHz). The 2 × 2 AMC structure can be further reduced to 76 × 76 mm^2^ (corresponding to 0.22∙λ_0_ × 0.22∙λ_0_ at 868 MHz) by cutting out the outer edges (Figure 5b), without degrading the complete structure performance. This size reduction is allowed because the coupling between the AMC and the tag is effective only in the region where they are overlapped. The reduction of the AMC structure causes an upward shift in the tag working frequency of about 80 MHz, as shown in Figure 5c, where we reported the comparison between the structure of Figure 5a and the structure of Figure 5b with the same values W_A1_ × L_A1_ for the NSSP aperture size. The tag working frequency can be moved back to the frequency of 868 MHz by simply adjusting the NSSP aperture size (from W_A1_ × L_A1_ to W_A2_ × L_A2_), achieving substantially the same behavior of the original AMC structure for the tag’s transmission coefficient τ, as apparent when looking at the red and green curves of Figure 5c.

**Figure 6:**
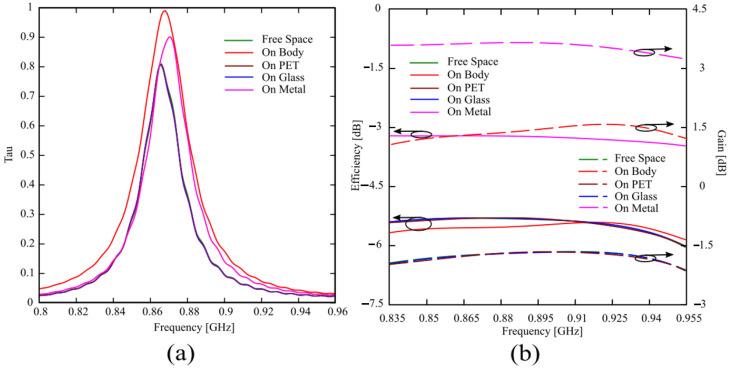
(**a**) Simulated transmission coefficient and (**b**) Gain and efficiency for the tag antenna attached on different materials.

**Figure 7:**
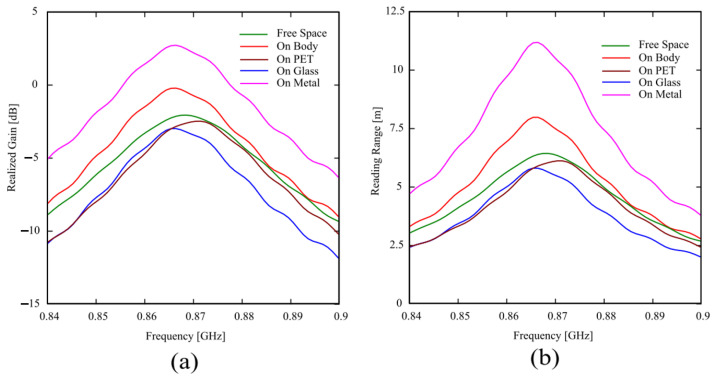
(**a**) Simulated realized gain and (**b**) reading range for the tag antenna attached on different materials.

The proposed tag on the AMC has been tested in free space and attached to different materials (the human body, a PET sheet, a glass sheet, and a metal plate). The PET has a dielectric permittivity equal to ε_r_ = 3 with tanδ = 0.002, the glass of ε_r_ = 4.82 with tanδ = 0.0054, and the metal plate is made of copper. The results reported in Figure 6 and Figure 7 show that the structure is platform tolerant and can be used indifferently to tag an arbitrary object, or in wearable applications. The tag on the AMC has been designed and tuned when attached to the human body; therefore, the simulated transmission coefficient reported in Figure 6a shows a little variation when considering a different platform (metal, glass, or PET), or when it is in free space. Moreover, the frequency response on the metal is very similar to the one with the tag attached to the human body (due to its high dielectric permittivity and losses), while the cases of free space, pet, and glass are almost indistinguishable, because of their low dielectric permittivity. Figure 6b shows the gain and efficiency of the tag attached to different materials; the simulated efficiency is almost the same for all the materials except for the metal case, where the efficiency is 2.5–3 dB higher; on the other hand, the gain of the tag in the free space or attached to glass or PET is almost the same, while it increases around 3 dB when the tag is attached to the human body, and a further 1.5–2 dB when it is placed onto a metal plate.

This is due to the shielding effect of both the human body and the metal plate, which generate reflected waves constructively adding to the incident ones in the broadside direction, improving the radiated field and gain.

Figure 7a shows the simulated realized gain of the tag, which has been computed as G_R_ = τ·G, and its behavior is consistent with the frequency responses of the transmission coefficient τ and gain G reported in Figure 6.

The theoretical read range reported in Figure 7b has been computed using the following expression [49]:

(1) $$r_{range}=\frac{\lambda }{4\pi }\sqrt{\frac{\frac{P_{CP}}{2}{\times G}_{t}{\times G}_{tag}\times \tau }{P_{chip}}}$$

where the transmitter power is equal to P_CP_ = 30 dBm (the reader antenna we will use for the measures radiates circular polarization) and G_t_ = 5.15 dB, whereas the read sensitivity of the IC Monza 4 equals to P_chip_ = −17.4 dBm. G_tag_ and τ are the same values obtained by CST Microwave Studio and reported in Figure 6. The obtained reading range is very good for each considered material on which the tag is attached, ranging from 5.5 m to 11 m for glass and metal, respectively. Again, according to the realized gains reported in Figure 7a, the read ranges for the tag attached to PET, glass, or in free space are very close, while the reading range increases about 2 m when the tag is attached to the human body, and doubles when it is attached on a metal plate.

**Figure 8:**
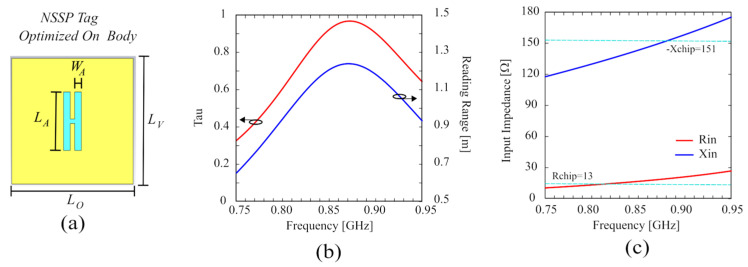
(**a**) Layout of the NSSP (without AMC support) optimized when attached to the phantom: L_O_ = 69.5 mm, L_V_ = 69.5 mm, L_A_ = 32.3 mm, W_A_ = 3.85 mm. (**b**) Simulated transmission coefficient and reading range. (**c**) Simulated input impedance.

**Figure 9:**
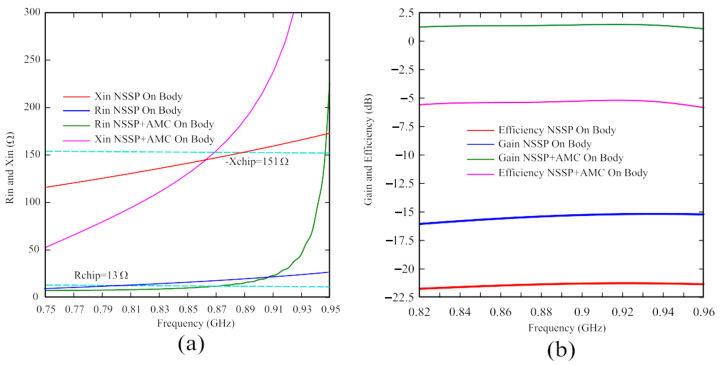
Comparison between the NSSP and the NSSP on the AMC structure attached to the phantom. (**a**) Simulated input impedance and (**b**) Gain and efficiency.

The performance of the proposed antenna has been compared with the NSSP antenna directly attached to the phantom to clearly highlight the significant improvement achieved through exploiting the isolation of the AMC structure. To obtain a fair comparison, since the NSSP behavior is strongly modified by coupling with the human body after removing the AMC layer, the NSSP antenna must be optimized for operation when directly attached to the phantom. The results of this optimization are reported in Figure 8, wherein the layout, transmission coefficient, reading range, and input impedance of the optimized NSSP are shown. As apparent from Figure 8a, the NSSP layout has been strongly modified in order to be matched to the body without the support of the AMC, and the aperture size has been significantly reduced. This matching provides a very good transmission coefficient of about 0.97 (see Figure 8b), with a good and relatively flat input impedance (Figure 8c); however, obviously, the ungrounded antenna shows bad performance in terms of reading range (with a peak of only 1.2 m, as reported in Figure 8b). In Figure 9, this optimized NSSP has been compared with the structure of Figure 5b, supported by the AMC. Figure 9a shows the input impedance, whereas in Figure 9b, the efficiency and the gain are reported. Although the matching is very good for both cases, the isolation given by the AMC structure allows one to obtain a considerable increment of around 17 dB in both gain and efficiency, resulting in a significant improvement of the wearable antenna performance. Moreover, the AMC structure allows an easy matching with a given microchip, requiring less significant changes to the NSSP aperture size to achieve an adequate value of the transmission coefficient.

The proposed structure can be tuned within the whole worldwide RFID band (from 868 to 960 MHz) by varying the length of the apertures of the tag, since the AMC behavior is maintained in this frequency band, as confirmed by the flat curves of gain and efficiency shown in Figure 6b. Moreover, our structure does not require the use of vias or shorting pins, nor lumped elements or parasitic elements to shrink down the resonant frequency. Finally, it achieves a read range of 5.5 m on low permittivity dielectric materials, of 8 m when attached to the human body, and of 11 m on a 200 × 200 mm^2^ metal plate using a low transmitting power P_t_ and a low gain G_t_ of the reader antenna if compared to the state-of-the-art structures, showing a good advantage with respect to the already published similar tag antennas, as summarized in Table 1.

## 4. Conclusions

The concept of AMC structures has been exploited to increase the isolation and robustness of standard wearable ungrounded antennas with respect to human body proximity. A platform-tolerant RFID tag has been designed, which can be easily tuned within the whole worldwide RFID band (from 868 to 960 MHz), using a relatively small AMC structure as a shielding element for an ungrounded RFID tag antenna. The proposed structure can be indifferently attached to low permittivity dielectric materials, metal objects, or the human body, achieving a read range of 5.5 m, 11 m, and 8 m, respectively. The isolation provided by the AMC planar structure increases the gain of the ungrounded RFID tag directly attached to the human body by about 13 dB, eases its matching with the microchip, and significantly reduces the undesired electromagnetic radiation toward the wearer. Finally, the designed tag exhibits very low manufacturing complexity and may be produced at a low cost.

## Figures and Tables

**Table 1:** Comparison with the state-of-the-art works in terms of dimensions and performance. L_x_ is the size along the x-axis, L_y_ is the size along the y-axis, P_tx_ is the power transmitted by the reader, G_tx_ is the gain of the reader antenna, P_chip_ is the sensitivity power of the tag microchip, G_tag_ is the gain of the tag, R_m_ is the tag’s measured reading range, R_t_ is the tag’s simulated reading range, and the letters M, B, and A indicate if the considered structure has been analyzed on metal, on the body, and if it is mounted on an AMC, respectively.

Ref.	L_x_ L_y_	H_z_	ε_r_	f_res_ (MHz)	P_tx_ (dBm)	G_tx_ (dBi)	P_chip_ (dBm)	G_tag_ (dBi)	R_m_ (m)	Rt (m)	M	B	A
[16]	100 × 60 mm^2^ (0.289 × 0.174 λ_0_^2^)	10 mm (0.029 λ_0_)	4.5	868	30	6	−17	-	12.2	-	Yes	No	Yes
[17]	100 × 20 mm^2^ (0.817 × 0.163 λ_0_^2^)	9.24 mm (0.075 λ_0_)	3.5	2450	32	6	−14	4.4	1	-	No	Yes	Yes
[18]	215 × 211 mm^2^ (0.656 × 0.644 λ_0_^2^)	6.4 mm (0.020 λ_0_)	4.4	915	36	-	−16.7	5	15.7	17.7	No	Yes	Yes
[19]	34.44 × 67 mm^2^ (0.105 × 0.204 λ_0_^2^)	3.63 mm (0.011 λ_0_)	6.45	915	33	-	−17	−2	4.8	-	Yes	No	Yes
[20]	44.1 × 44.1 mm^2^ (0.323 × 0.323 λ_0_^2^)	1.524 mm (0.011 λ_0_)	3.28	2200	-	-	-	2.9	-	-	Yes	No	Yes
[21]	25 × 25 mm^2^ (0.072 × 0.072 λ_0_^2^)	2.5 mm (0.007 λ_0_)	9	868	35.2	-	−18.5	-	0.98	1.3	Yes	No	No
[22]	40 × 40 mm^2^ (0.122 × 0.122 λ_0_^2^)	1.6 mm (0.005 λ_0_)	3.3	915	36	-	−19.9	−5.5	7.7	-	Yes	No	No
[23]	30 × 30 mm^2^ (0.092 × 0.092 λ_0_^2^)	1.6 mm (0.005 λ_0_)	1.06	915	36	-	−19.9	−12	3.5	-	Yes	No	No
[24]	104 × 31 mm^2^ (0.301 × 0.090 λ_0_^2^)	7.6 mm (0.022 λ_0_)	4.4	868	36	-	−18.5	1.5	11.8	-	Yes	No	No
[25]	64 × 64 mm^2^ (0.195 × 0.195 λ_0_^2^)	2 mm (0.005 λ_0_)	4.4	915	36	-	−17	-	10.2	12	Yes	No	No
[26]	50 × 50 mm^2^ (0.153 × 0.153 λ_0_^2^)	2 mm (0.006 λ_0_)	4.4	915	36	6	−19.5	−4.9	8.5	-	Yes	No	No
[27]	56 × 56 mm^2^ (0.171 × 0.171 λ_0_^2^)	0.4 mm (0.0012 λ_0_)	4.6	915	36	9	−14	1.4	9.9	10	No	No	No
[28]	58 × 58 mm^2^ (0.177 × 0.177 λ_0_^2^)	1.6 mm (0.005 λ_0_)	4.4	915	36	8	−17.4	1.28	15.6	15	No	No	No
[29]	35.6 × 35.6 mm^2^ (0.109 × 0.109 λ_0_^2^)	0.508 mm (0.0015 λ_0_)	3.38	915	35.2	14	−15	-	7.6	-	No	No	No
[30]	58.6 × 58.6 mm^2^ (0.181 × 0.181 λ_0_^2^)	0.4 mm (0.0012 λ_0_)	4.4	925	30	9	−17	1.7	20.5	19.9	No	No	No
This work	76 × 76 mm^2^ (0.22 × 0.22 λ_0_^2^)	3.245 mm (0.009 λ_0_)	6	868	30	5.16	−17.3	0.7	-	11	Yes	Yes	Yes

## Data Availability

Data are contained within the article.

## References

[1] Hall P.S., Hao Y., Nechayev Y.I., Alomainy A., Constantinou C.C., Parini C., Kamarudin M.R., Salim T.Z., Hee D.T.M., Dubrovka R. (2007). Antennas and propagation for on-body communication systems. IEEE Antennas Propag. Mag..

[2] Serra A.A., Nepa P., Manara G. (2012). A Wearable Two-Antenna System on a Life Jacket for Cospas-Sarsat Personal Locator Beacons. IEEE Trans. Antennas Propag..

[3] Michel A., Karathanasis K., Nepa P., Volakis J.L. (2015). Accuracy of a multi-probe conformal sensor in estimating the dielectric constant in deep biological tissues. IEEE Sens. J..

[4] Marrocco G. (2007). RFID antennas for the UHF remote monitoring of human subjects. IEEE Trans. Antennas Propag..

[5] Kiourti A., Lee C., Volakis J.L. (2016). Fabrication of textile antennas and circuits with 0.1 mm precision. IEEE Antennas Wirel. Propag. Lett..

[6] Moro R., Agneessens S., Rogier H., Dierck A., Bozzi M. (2015). Textile microwave components in substrate integrated waveguide technology. IEEE Trans. Microw. Theory Techn..

[7] Casula G.A., Michel A., Montisci G., Nepa P., Valente G. (2017). Energy-based considerations for ungrounded wearable UHF antenna design. IEEE Sens. J..

[8] Casula G.A., Michel A., Nepa P., Montisci G., Mazzarella G. (2016). Robustness of wearable UHF-band PIFAs to human-body proximity. IEEE Trans. Antennas Propag..

[9] Michel A., Colella R., Casula G.A., Nepa P., Catarinucci L., Montisci G., Mazzarella G., Manara G. (2018). Design considerations on the placement of a wearable UHF-RFID PIFA on a compact ground plane. IEEE Trans. Antennas Propag..

[10] Casula G.A., Montisci G., Valente G., Gatto G. (2018). A robust printed antenna for UHF wearable applications. IEEE Trans. Antennas Propag..

[11] Casula G.A., Montisci G. (2019). A design rule to reduce the human body effect on wearable PIFA antennas. Electronics.

[12] Casula G.A., Montisci G., Rogier H. (2020). A wearable textile RFID tag based on an eighth-mode substrate integrated waveguide cavity. IEEE Access.

[13] Zhu S., Langley R. (2009). Dual-band wearable textile antenna on an EBG substrate. IEEE Trans. Antennas Propag..

[14] Kim S., Ren Y.J., Lee H., Rida A., Nikolaou S., Tentzeris M.M. (2012). Monopole antenna with inkjet-printed EBG array on paper substrate for wearable applications. IEEE Antennas Wirel. Propag. Lett..

[15] Raad H.R., Abbosh A.I., Al-Rizzo H.M., Rucker D.G. (2013). Flexible and compact AMC based antenna for telemedicine applications. IEEE Trans. Antennas Propag..

[16] Park I.Y., Kim D. (2014). Artificial magnetic conductor loaded long range passive RFID tag antenna mountable on metallic objects. Electron Lett..

[17] Sanusi O.M., Ghaffar F.A., Shamim A., Vaseem M., Wang Y., Roy L. (2019). Development of a 2.45 GHz Antenna for Flexible Compact Radiation Dosimeter Tags. IEEE Trans. Antennas Propag..

[18] Hong J.H., Chiu C.-W., Wang H.-C. (2018). Design of Circularly Polarized Tag Antenna with Artificial Magnetic Conductor for on-Body Applications. Prog. Electromagn. Res. C.

[19] Kim D., Yeo J. (2008). Low-Profile RFID Tag Antenna Using Compact AMC Substrate for Metallic Objects. IEEE Antennas Wirel. Propag. Lett..

[20] De Cos M.E., Las-Heras F. (2012). Dual-Band Antenna/AMC Combination for RFID. Int. J. Antenn. Propag..

[21] Michel A., Franchina V., Nepa P., Salvatore A. (2019). A UHF RFID Tag Embeddable in Small Metal Cavities. IEEE Trans. Antennas Propag..

[22] Yang E.S., Son H.W. (2016). Dual-polarised metal-mountable UHF RFID tag antenna for polarisation diversity. Electron. Lett..

[23] Bong F.-L., Lim E.-H., Lo F.-L. (2018). Compact orientation insensitive dipolar patch for metal-mountable UHF RFID tag design. IEEE Trans. Antennas Propag..

[24] Hamani A., Yagoub M.C.E., Vuong T.-P., Touhami R. (2017). A Novel Broadband Antenna Design for UHF RFID Tags on Metallic Surface Environments. IEEE Antennas Wirel. Propag. Lett..

[25] Ng W.-H., Lim E.-H., Bong F.-L., Chung B.-K. (2020). Compact Folded Crossed-Dipole for On-Metal Polarization Diversity UHF Tag. IEEE J. Radio Freq. Identif..

[26] Althobaiti T., Sharif A., Ouyang J., Ramzan N., Abbasi Q.H. (2020). Planar Pyramid Shaped UHF RFID Tag Antenna with Polarisation Diversity for IoT Applications Using Characteristics Mode Analysis. IEEE Access.

[27] Inserra D., Wen G. (2019). Compact crossed dipole antenna with meandered series power divider for UHF RFID tag and handheld reader devices. IEEE Trans. Antennas Propag..

[28] Bhaskar S., Singh A.K. (2019). Linearly tapered meander line cross dipole circularly polarized antenna for UHF RFID tag applications. Int. J. RF Microw. Comput. Aided Eng..

[29] Tran H.H., Ta S.X., Park I. (2015). A compact circularly polarized crossed dipole antenna for an RFID tag. IEEE Antennas Wirel. Propag. Lett..

[30] Chen H.-D., Sim C.-Y.-D., Tsai C.-H., Kuo C. (2016). Compact circularly polarized meandered-loop antenna for UHF-band RFID tag. IEEE Antennas Wirel. Propag. Lett..

[31] Hsu H.-T., Huang T.-J. (2014). A 1 × 2 Dual-Band Antenna Array for Radio-Frequency Identification (RFID) Handheld Reader Applications. IEEE Trans. Antennas Propag..

[32] Sharif A., Kumar R., Althobaiti T., Alotaibi A.A., Safi L., Ramzan N., Imran M.A., Abbasi Q.H. (2023). Bio-Inspired Circular-Polarized UHF RFID Tag Design Using Characteristic Mode Analysis. IEEE Sens. J..

[33] Romputtal A., Phongcharoenpanich C. (2019). IoT-Linked Integrated NFC and Dual Band UHF/2.45 GHz RFID Reader Antenna Scheme. IEEE Access.

[34] Hammad H.F. (2021). New Technique for Segmenting RFID Bandwidth for IoT Applications. IEEE J. Radio Freq. Identif..

[35] Anee R.-E.-A., Karmakar N.C. (2013). Chipless RFID Tag Localization. IEEE Trans. Microw. Theory Tech..

[36] Cappelli I., Fort A., Mugnaini M., Panzardi E., Pozzebon A., Tani M., Vignoli V. (2021). Battery-Less HF RFID Sensor Tag for Soil Moisture Measurements. IEEE Trans. Instrum. Meas..

[37] Lasantha L., Karmakar N.C., Ray B. (2023). Chipless RFID Sensors for IoT Sensing and Potential Applications in Underground Mining—A Review. IEEE Sens. J..

[38] Alam M.S., Misran N., Yatim B., Islam M.T. (2013). Development of electromagnetic band gap structures in the perspective of microstrip antenna design. Int. J. Antenn. Propag..

[39] Liu Z.-G., Ge Z.-C., Chen X.-Y. (2009). Research progress on Fabry-Perot resonator antenna. J. Zhejiang Univ. Sci. A.

[40] Bala B.D., Rahim M.K.A., Murad N.A. (2014). Complementary electric-LC resonator antenna for WLAN applications. Appl. Phys. A.

[41] Alu A., Engheta N. (2004). Guided modes in a waveguide filled with a pair of Single-Negative (SNG), Double-Negative (DNG), and/or Double-Positive (DPS) layers. IEEE Trans Microw Theory Techniq..

[42] Cheribi H., Ghanem F., Kimouche H. (2013). Metamaterial-based frequency reconfigurable antenna. Electron. Lett..

[43] Jose J. (2014). Frequency selective bistable switching in metamaterial based photonic bandgap medium. Opt. Commun..

[44] Hu F., Zou T., Quan B., Xu X., Bo S., Chen T., Wang L., Gu C., Li J. (2014). Polarization-dependent terahertz metamaterial absorber with high absorption in two orthogonal directions. Opt. Commun..

[45] Nouh M., Aldraihem O., Baz A. (2014). Vibration characteristics of metamaterial beams with periodic local resonances. J. Vib. Acoust..

[46] GuoHe W., Li L., Teng B.T., Sun X. (2012). A wideband and dual resonant terahertz metamaterial using a modified SRR structure. Prog. Electromagn. Res..

[47] Costa F., Monorchio A. (2010). Multiband electromagnetic wave absorber based on reactive impedance ground planes. IET Microw. Antenn. Propag..

[48] Genovesi S., Monorchio A., Mittra R., Manara G. (2007). A sub-boundary approach for enhanced particle swarm optimization and its application to the design of artificial magnetic conductors. IEEE Trans. Antennas Propag..

[49] Rao K., Nikitin P., Lam S. (2005). Antenna design for UHF RFID tags: A review and a practical application. IEEE Trans. Antennas Propag..

